# Berberine Facilitates Extinction of Drug-Associated Behavior and Inhibits Reinstatement of Drug Seeking

**DOI:** 10.3389/fphar.2020.00476

**Published:** 2020-04-24

**Authors:** Xi Shen, Rongji Hui, Yixiao Luo, Hailei Yu, Suiyuan Feng, Bing Xie, Haitao Bi, Ewa Galaj, Bin Cong, Chunling Ma, Di Wen

**Affiliations:** ^1^College of Public Health, Hebei Medical University, Shijiazhuang, China; ^2^College of Forensic Medicine, Hebei Key Laboratory of Forensic Medicine, Collaborative Innovation Center of Forensic Medical Molecular Identification, Hebei Medical University, Shijiazhuang, China; ^3^Key Laboratory of Molecular Epidemiology of Hunan Province, School of Medicine, Hunan Normal University, Changsha, China; ^4^Molecular Targets and Medication Discovery Branch, National Institute on Drug Abuse, Baltimore, MD, United States

**Keywords:** drug addiction, relapse, extinction, drug-associated memory, berberine

## Abstract

A high rate of relapse is a major clinical problem among drug-addicted individuals. Persistent traces of drug-associated reward memories contribute to intense craving and often trigger relapse. A number of interventions on drug-associated memories have shown significant benefits in relapse prevention. Among them are pre- or post-extinction pharmacological manipulations that facilitate the extinction of drug-associated behavior. Berberine, a bioactive isoquinoline alkaloid, has been recently reported to provide therapeutic benefits for a number of central nervous system (CNS) disorders, including morphine addiction. The present study aimed to investigate whether berberine could serve as a post-extinction pharmacological intervention agent to reduce risks of reinstatement of drug seeking. We found that an intragastric administration of berberine at doses of 25 and 50 mg/kg during the critical time window significantly facilitated the extinction of morphine-reward related behavior in free access and confined conditioned place preference (CPP) extinction paradigms, and subsequently, it prevented reinstatement and spontaneous recovery of morphine-induced CPP in mice. Intriguingly, the berberine treatment with or without extinction training altered expression of plasticity-related proteins such as brain-derived neurotrophic factor (BDNF), AMPA receptors (GluA1 and GluA2) in the nucleus accumbens (NAc). Moreover, the post-extinction berberine treatment significantly reduced reinstatement of cocaine-induced CPP and operant intravenous self-administration (IVSA) memories in rats. Altogether, our findings suggest that extinction training combined with the post-extinction berberine treatment can facilitate extinction of drug-associated behavior making it an attractive therapeutic candidate in relapse prevention.

## Introduction

Drug addiction is a serious problem that poses numerous hazards on public health and social welfare worldwide, including China (West et al., 2019). High rates of relapse, which are often triggered by exposure to drug-associated cues even after extended periods of abstinence, are the greatest clinical concern (Grant et al., 2010; Dong et al., 2017). Converging evidence suggests that drug addiction is based on persistent drug reward memory traces that are formed in the brain during drug-induced euphoric effects and exposure to drug-associated cues; both of which play a critical role in relapse (Kelley, 2004; Milton and Everitt, 2012; Tronson and Taylor, 2013).

Drug-associated memories share the same processes as non-drug related memories, that is, conditioning/acquisition, consolidation, retrieval, reconsolidation, extinction, and storage (Kelley, 2004). Extinction is an active process that leads to a progressive decline in acquired responses and involves formation of new memories, which temporally suppresses the expression of original drug-associated memories (Marlatt, 1990; Millan et al., 2011; McNally, 2014). In the ongoing search for novel therapeutics that would facilitate the extinction of drug-associated behavior, recent studies have provided favorable outcomes in various animal models of addiction (Havermans and Jansen, 2003; Kelamangalath et al., 2007; Xue et al., 2012; Luo et al., 2015). In the extinction therapy, addicted individuals are repeatedly exposed to drug-associated cues in the absence of drugs, in order to inhibit craving, rewarding effects of drug cues and prevent relapse. Moreover, a growing number of studies have demonstrated that many pre- or post-extinction pharmacological interventions can facilitate extinction of drug-associated behavior (Zhou and Kalivas, 2008; Ma et al., 2012; Chesworth and Corbit, 2017; Liu et al., 2019); therefore, they can reduce the propensity to relapse.

Berberine, a bioactive isoquinoline alkaloid that can be extracted from berberidaceae family plants *Berberis*, was first used in China as a traditional medicine by Shennong around 3000 BC (Tang et al., 1962). Since then, berberine has been used as a common over-the-counter (OTC) medication for gastrointestinal infections but recent studies report a variety of its therapeutic benefits including a reduction in blood sugar levels (Trimarco et al., 2015), weight loss (Zhang et al., 2014), and improvement in heart health (Allijn et al., 2017) as well as anxiolytic, anti-depressant, anti-psychotic, anti-convulsant, and anti-amnesic effects (Kulkarni and Dhir, 2008; Kulkarni and Dhir, 2010; de Oliveira et al., 2016; Huang et al., 2018). The inhibitory effects of berberine on morphine-induced sensitization (Yoo et al., 2006), morphine withdrawal-associated behavior (Lee et al., 2012), and acquisition and reinstatement of morphine-induced conditioned place preference (CPP) (Vahdati Hassani et al., 2016) have also been reported. However, the exact mechanisms of action by which berberine produces its therapeutic effects remain to be elucidated. Furthermore, it is unknown whether berberine could serve as a post-extinction pharmacological intervention to diminish original drug-related memories and propensity to relapse.

Recent studies focused on understanding the circuitry and cellular mechanisms underlying extinction of drug-associated behavior have provided compelling evidence for the involvement of glutamatergic, dopaminergic, and noradrenergic plasticity in the nucleus accumbens (NAc), amygdala (Amy), and prefrontal cortex (PFC) (Torregrossa et al., 2010; Gass and Chandler, 2013; Goode and Maren, 2019; Zhang et al., 2019). Some of the extinction-related neuroplastic changes involve brain-derived neurotropic factor (BDNF) and AMPA receptor (GlueA1 and GluA2) (Xue et al., 2014), both of which are deemed for their critical roles in long-term memory and drug addiction (Cleva et al., 2010; Barker et al., 2015). Furthermore, there is evidence that manipulation of extinction-induced plasticity involving the upregulation of BDNF and AMPA receptor expression could boost the extinction procedure and diminish drug-seeking (Sutton et al., 2003; Briand et al., 2012). However, it is unknown whether berberine implemented after the extinction procedure can produce similar neuroplastic changes in the BDNF and AMPA receptor expressions.

Reinstatement of drug-induced CPP and drug seeking in the intravenous self-administration (IVSA) paradigm are most commonly used animal models that elegantly mimic the human experience of abstinence and relapse. Two types of CPP extinction training paradigms, involving either confinement of an animal to a drug-paired context *or* providing free access to drug-paired and non–drug-paired contexts, are often used to extinguish drug-associated seeking behaviors (Calcagnetti and Schechter, 1993; Mueller and Stewart, 2000). Herein, using both types of CPP extinction paradigms, we investigated potential effects of the berberine treatment, administered during the critical time window of extinction memory consolidation, on extinction of morphine-induced drug-associated behaviors. To test its reproducibility, we examined the effects of the berberine treatment on extinction of cocaine reward-related behaviors using the CPP and IVSA paradigms. The original drug-associated memory was subsequently assessed in a drug-primed reinstatement test and in a spontaneous recovery test with the passage of time. Furthermore, the associated changes in accumbal BDNF and AMPA receptor (GluA1 and GluA2) expression in animals undergoing the post-extinction berberine treatment were explored.

## Materials and Methods

### Animals

A total of 178 male C57BL/6 mice (initially weighing 20–22 g) and 30 male Sprague-Dawley rats (initially weighing 220–240 g) were purchased from Beijing Vital River Laboratory Animal Technology Co. Ltd, China. Animal care, and the experimental procedures were conducted in accordance with the Animal Research: Reporting of In Vivo Experiments (ARRIVE) guidelines and were carried out in accordance with the National Institutes of Health guide for the care and use of Laboratory animals (NIH Publications No. 8023, revised 1978). Animals were housed in a climate-controlled environment with a constant temperature (23 ± 2°C), humidity (approximately 60%), and a 12-h light/dark cycle (lights on at 7:00 am) were maintained. Food and water were available *ad libitum*. All animals were acclimated to the laboratory housing conditions for at least 5 days before the start of experimentation. The Local Committee on Animal Care and Use and Protection of the Hebei Medical University approved the experimental procedures.

### Drugs

Morphine sulfate and cocaine hydrochloride were purchased from Qinghai Pharmaceutical Factory (Xining, China), and both were dissolved in 0.9% physiological saline. Concentrations of morphine and cocaine were adjusted to an appropriate injection volume of 1 mL/kg body weight immediately before use. Berberine was purchased from J&K Chemical Ltd., Shanghai, China, and resuspended to concentrations of 12.5, 25, and 50 mg/mL in corn oil immediately before use. Animals in the control group were treated with intragastric administration of the corresponding vehicle (corn oil,1 mL/kg).

### Behavioral Measurements

#### Conditioned Place Preference

The conditioned place preference (CPP) test was conducted in an apparatus composed of three compartments that could be isolated by guillotine doors. All three chambers were visually and tactually distinct. One chamber (15 × 15 × 30 cm) had white walls with 3 cm wide horizontal black stripes and a floor with rough polyvinyl chloride mesh (0.8 × 0.8 cm), while the other chamber (15 × 15 × 30 cm) had black walls with 3 cm wide vertical white stripes and a floor with smooth polyvinyl chloride mesh (0.8 × 0.8 cm). Two identically sized compartments were connected by a narrower chamber (5 × 15 × 30 cm) with gray walls and a stainless-steel mesh floor. Animal behavior during a CPP test was recorded automatically using the SMART video tracking system (Panlab Technology for Bioresearch, Spain).

The unbiased CPP paradigm consisted of the following phases: pre-conditioning, conditioning, post-conditioning, extinction, reinstatement, and spontaneous recovery. In each CPP test, the mouse was placed in the middle compartment with the guillotine doors removed to allow free access to the entire apparatus for 15 min. CPP score (s) was calculated as the time spent in the drug-paired chamber minus the time spent in the saline-paired chamber during the test.

##### Pre-Conditioning

The first CPP test (day 1) was performed without any drug treatment to evaluate the initial unconditioned preference. Mice with CPP scores greater than 150 s were considered to have a chamber bias and were excluded from the study.

##### Conditioning

A place conditioning phase started on Day 2. Mice received an intraperitoneal injection of either morphine (10 mg/kg), cocaine (10 mg/kg), or saline (1 mL/kg) and were immediately confined to one of the compartments for 45 min. Each mouse received six morphine or cocaine conditioning sessions and during alternating sessions six saline pairings (days 2–7). These sessions were conducted twice a day at 8:00 and 14:00. Drug-paired compartments were assigned in a counterbalanced fashion such that in each group, approximately half mice were conditioned with morphine (or cocaine) to one compartment, and the rest were conditioned to the other compartment.

##### Post-Conditioning

The second CPP test was conducted in the post-conditioning phase (day 8), during which no drug treatment was administered, and animals were allowed to move freely across all three compartments for 15 min. In order to test the extinction of drug-associative preference behavior, mice with CPP scores less than 200 s were considered to have no preference and were excluded from further study.

##### Extinction

Following drug conditioning, the extinction of CPP phase occurred on days 9 to 14 involving two different procedures: free access to all compartments or confinement to the drug-paired context. Animals assigned to the extinction procedure with free access underwent repeated 15-min CPP tests for 6 days, while mice in the confined exposure procedure experienced alternative confinement to morphine-/cocaine- and saline-paired compartments for 45 min after receiving an intraperitoneal injection of saline (1 mL/kg). Berberine (12.5, 25, and 50 mg/kg, i.g.) or corn oil (1 mL/kg, i.g.) was administered to mice 1 h after each extinction training session. Moreover, memories are known to become impervious to interferences that disrupt synaptic consolidation within 6 h after the training (Dudai, 2004). To test whether berberine can target the consolidation of extinction memories within or beyond the critical time window, the berberine treatment was administered 1 h or 6 h after each extinction session to mice with free access CPP extinction training.

##### Reinstatement and Spontaneous Recovery

The extinguished animals were subjected to the reinstatement or spontaneous recovery phase. During the reinstatement test, animals received a systemic injection of saline (1 mL/kg, i.p.), followed by a priming injection of morphine (3 mg/kg, i.p.) or cocaine (5 mg/kg, i.p.) 10 min before the CPP test on days 15 and 16, respectively. CPP was considered as extinguished when no significant difference between the preference scores of saline priming test and pre-test was observed. Locomotor activity during morphine priming test was recorded by the SMART software (Panlab Technology for Bioresearch, Spain) (data were shown in **Supplementary Files**, **Figure S1**). During the spontaneous recovery test, the preference score was assessed 2, 3, and 4 weeks after the last extinction training session (Days 28, 35, and 42).

#### Intravenous Self-Administration

A cocaine-induced IVSA training was conducted in accordance with methods employed in the study of Xue et al. (2012). Rats weighing 250 to 280 g were anaesthetized with isoflurane and implanted with a single silicon catheter in the right jugular vein. Catheters were flushed daily with 0.2 mL saline solution containing gentamycin (0.5 mg/mL) and heparin (30 U/mL). Rats were housed individually after the catherization surgery and were allowed to recover for 5 days before the behavioral training began.

##### IVSA Training

Rats were trained to intravenously self-administer cocaine (0.75 mg/kg/infusion) under a fixed ratio (FR1) schedule of reinforcement during 3 h sessions for 10 days. Each operant chamber (30 × 30 × 40 cm) was equipped with two nose-poke operandi located 9 cm above the chamber floor (AniLab Software and Instruments, Ningbo, China). Each infusion was accompanied with the illumination of the light and tone cues for 5 s, followed by 40 s time out. Inactive nose-pokes were also recorded; however, they had no programmed consequences. Drug infusions were given at most 20 times per hour.

##### IVSA Extinction

The extinction procedure was same as the one used during the IVSA training, with the exception that drug was no longer available. Active lever presses resulted in an infusion of saline and the presentation of the light and tone cues simultaneously. Rats experienced 3 h daily extinction sessions for 10 days (days 11–20). The extinction criterion was defined as a decrease in the number of active nose-pokes during two consecutive sessions to < 20% of mean nose-pokes counted in the last 3 days of cocaine IVSA. Berberine (12.5, 25, and 50 mg/kg, i.g.) or corn oil (1 mL/kg, i.g.) was administered to rats at 1 h after each extinction training session.

##### Reinstatement Test

During the reinstatement test, rats received a control injection of saline (1 mL/kg, i.p.) or a priming injection of cocaine (5 mg/kg, i.p.) 10 min before the test on Day 21. The conditions of the reinstatement test were similar to the ones of IVSA training. The test session began with the illumination of the light, which remained on throughout the remaining session time. Active nose pokes during the test resulted in activation of the tone-light cues that were previously paired with drug infusions. Active and inactive nose pokes during the 3-h test were counted.

### Western Blot

Mice from morphine CPP/free access extinction procedure were sacrificed by decapitation at 1 h after the last extinction training and drug treatment, and the NAc tissues of five animals from each group were harvested. A Western blot assay was performed to evaluate changes in BDNF, GluA1, and GluA2 levels in the NAc of mice. Equal amounts of denatured total protein (10 µg) from each sample were separated using 12% SDS-PAGE. The separated proteins were transferred onto a 0.2 µm nitrocellulose membrane. The membrane was blocked with 5% non-fat dry milk in a TBS solution and probed with specific rabbit polyclonal GluA1 (ab31232) antibody (diluted 1:1000, Abcam), rabbit monoclonal GluA2 (ab206293) antibody (diluted 1:1000, Abcam), rabbit monoclonal BDNF (ab108319) antibody (diluted 1:1000, Abcam) overnight at 4°C. The membrane was then washed and incubated with fluorophore-conjugated donkey anti-rabbit or goat anti-mouse secondary antibody (diluted 1:10000, Rockland). The secondary antibody was light-excited (700/800 nm wavelength), and the emitted light was detected and analyzed using an Odyssey gel imaging system (LI-COR, Inc., Lincoln, NE, USA). GAPDH expression was analyzed in the same blot using a specific mouse polyclonal antibody (diluted 1:5000, ABclonal). Image J Program Analyzer (Media Cybernetics, USA) was used to quantify the protein band density.

### Data Analysis

Data are presented as means ± SEM. Statistical analyses were performed separately for CPP or IVSA training, extinction training, drug-priming-induced reinstatement, and spontaneous recovery. Paired Student’s *t* test was applied to compare the pre-test and post-test CPP scores from the CPP training procedure. The results of the CPP (CPP scores) and IVSA (nose-pokes) tests were analyzed using separate two-way ANOVAs (mixed design), with test phase or reinstatement condition as within-subjects factor and the berberine treatment as between-subjects factor. BDNF, GluA1, and GluA2 expressions were analyzed using separate two-way ANOVAs. *Post hoc* comparisons in the ANOVA were performed using Bonferroni test. The threshold for statistical significance was *p* < 0.05 (GraphPad, v.8.0, California, USA).

## Results

### The Effect of Berberine on Extinction of Morphine-Induced Drug-Associated Behavior in a Free Access CPP Extinction Paradigm

**Figure 1:**
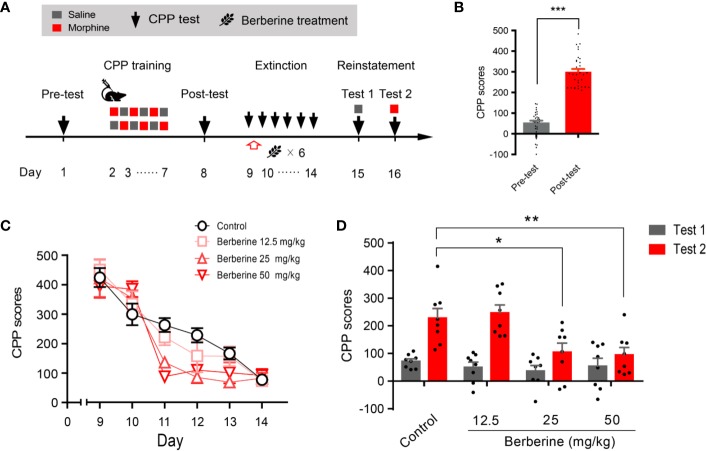
The effect of berberine on extinction of morphine-induced drug-associated behavior in free access CPP extinction paradigm **(A)** The experimental timeline of behavioral training and tests, and drug treatments **(B)** CPP scores of pre-test and post-test, which were conducted before and after CPP training. Significant place preference was observed after morphine-paired CPP training. n = 32; ***p < 0.001, compared to the pre-test **(C)** Extinction of morphine-induced drug-associated behavior in a free access CPP extinction paradigm. Corn oil (1 mL/kg, i.g.) and berberine (12.5, 25, and 50 mg/kg, i.g.) were administered to mice in the control and berberine treatment groups, respectively, 1 h after each extinction training session **(D)** Reinstatement of morphine-induced CPP. A priming injection of saline (1 mL/kg, i.p.) and morphine (3 mg/kg, i.p.) was administered 10 min before the reinstatement tests 1 and 2, respectively. n = 8 for each group; *p < 0.05, **p < 0.01, compared to the control group. Data are expressed as mean ± SEM. CPP, conditioned place preference.

Training, tests, and drug treatments were conducted as per the timeline shown in **Figure 1A**. In the pre-conditioning and conditioning sessions, 8 out of 40 mice were excluded due to initial chamber bias or training failure. As shown in **Figure 1B**, a total of 32 mice showed significant difference in place preference (t _31_ = 12.80, *p* < 0.001) after morphine-conditioning. These animals were subsequently divided into four independent groups (n = 8 per group) and reintroduced into free access CPP extinction paradigm. Corn oil (1 mL/kg, i.g.) and berberine (12.5, 25, and 50 mg/kg, i.g.) were administered to mice in the control and berberine treatment groups, respectively, at 1 h after each extinction training session for 6 days. A two-way ANOVA (repeated measures) revealed a significant difference in the CPP scores (**Figure 1C**) across berberine dose (F_(3,__28)_ = 3.723, *p* = 0.023), extinction sessions (F_(5,__140)_ = 75.26, *p* < 0.001), and berberine dose × extinction sessions interactions (F_(15,__140)_ = 2.084, *p* = 0.014). Thus, our data indicate that the berberine treatment can facilitate the extinction of morphine-induced drug-associated behavior in free access CPP extinction paradigm.

During the reinstatement session, a priming injection of saline (1 mL/kg, i.p.) or morphine (3 mg/kg, i.p.) was administered 10 min before the reinstatement tests 1 and 2 (**Figure 1A**), respectively. A two-way ANOVA (mixed design) revealed a significant difference in the CPP scores across reinstatement test phase (F_(1,__28)_ = 30.19, *p* < 0.001), berberine dose (F_(3,__28)_ = 8.563, *p* < 0.001), and berberine dose × reinstatement test phase interactions (F_(3,__28)_ = 3.023, *p* = 0.046). Bonferroni *post hoc* analysis indicated significant inhibitory effects of berberine at doses of 25 mg/kg (*p* < 0.05) and 50 mg/kg (*p* < 0.01), but not 12.5 mg/kg (*p* > 0.05) on morphine-primed reinstatement of preference for morphine-paired compartment (**Figure 1D**). Therefore, the berberine treatment delivered as a post-extinction intervention also dose-dependently inhibited a drug-primed reinstatement of morphine CPP.

### The Effect of Berberine on Extinction of Morphine-Induced Drug-Associated Behavior in a Confined CPP Extinction Paradigm

**Figure 2:**
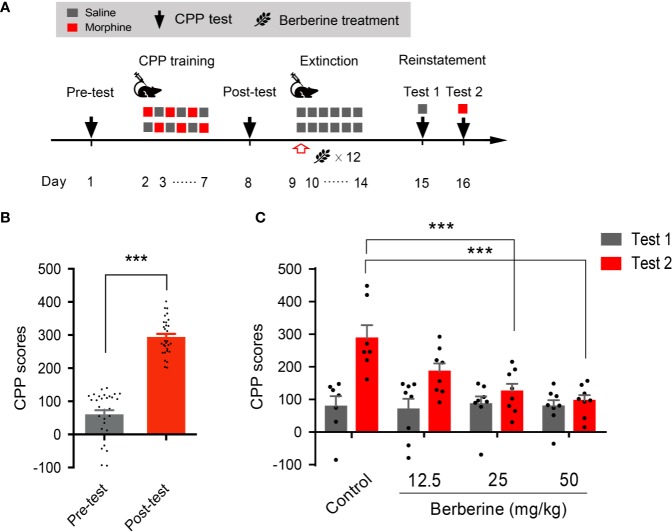
The effect of berberine on extinction of morphine-induced drug-associated behavior in a confined CPP extinction paradigm **(A)** The experimental timeline of the behavioral training and tests, and drug treatments **(B)** CPP scores of pre-test and post-test, which were conducted before and after CPP training. n = 31; ***p < 0.001, compared to the pre-test **(C)** Reinstatement of morphine-induced original drug-associated memory after the confined CPP extinction training procedure. Corn oil (1 mL/kg, i.g.) and berberine (12.5, 25, and 50 mg/kg, i.g.) were administered 1 h after each extinction training session. A priming injection of saline (1 mL/kg, i.p.) and morphine (3 mg/kg, i.p.) was administered 10 min before the reinstatement tests 1 and 2, respectively. n = 7–8 per group; ***p < 0.001, compared to the control group. Data are expressed as mean ± SEM.

We also used a confined CPP extinction paradigm (**Figure 2A**) to elucidate the effect of berberine on extinction of morphine-induced drug-associated behavior. Nine out of 40 mice were excluded from the study due to strong initial compartment bias or training failure. The CPP scores in the post-conditioning test were analyzed for 31 mice (t _30_ = 16.64, *p* < 0.001, **Figure 2B**). Next, these mice were assigned to four independent groups (n = 7, 8, 8, and 8), and were subjected to 6 morphine conditioning sessions, each alternated with saline-conditioning (saline at 1 mL/kg, i.p.). Corn oil (1 mL/kg, i.g.) and berberine (12.5, 25, and 50 mg/kg, i.g.) were administered at 1 h after each extinction session to mice in the control and berberine groups, respectively. On days 14 and 15, a priming injection of saline (1 mL/kg, i.p.) or morphine (3 mg/kg, i.p.) was delivered 10 min before the reinstatement tests 1 and 2 (**Figure 2A**). A two-way ANOVA (mixed design) revealed a significant difference in the CPP scores (**Figure 2C**) across berberine dose (F_(3,__27)_ = 5.076, *p* = 0.007), reinstatement test phase (F_(1,__27)_ = 22.69, *p* < 0.001), and berberine dose × reinstatement test phase interaction (F_(3,__27)_ = 4.512, *p* = 0.011). A subsequent Bonferroni *post hoc* analysis showed that berberine at the doses of 25 mg/kg (*p* < 0.001) and 50 mg/kg (*p* < 0.001) significantly decreased the CPP scores during morphine-primed reinstatement. No difference was revealed between the 12.5 mg/kg berberine and control groups (*p* > 0.05). These results provide additional evidence that berberine can facilitate extinction of drug-associated behavior, leading to a reduction in reinstatement of morphine CPP.

### Time Window Determines the Effect of Berberine on Extinction of Morphine-Induced Drug-Associated Behavior

**Figure 3:**
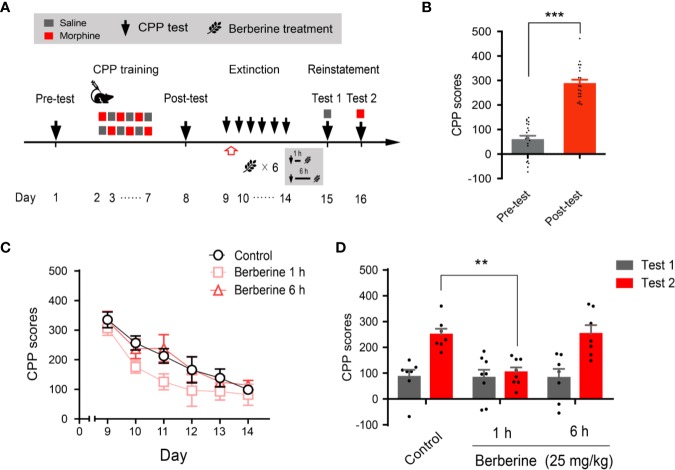
The critical time window determines the effect of berberine treatment on extinction of morphine-induced drug-associated behavior **(A)** The experimental timeline of behavioral training and tests, and drug treatments **(B)** CPP scores of pre-test and post-test, which were conducted before and after CPP training. n = 22; ***p < 0.001, compared to the pre-test **(C)** Extinction of morphine-induced drug-associated memory in a CPP extinction paradigm. Berberine (25 mg/kg, i.g.) was administered 1 h or 6 h after each extinction training session **(D)** Reinstatement of morphine-induced drug-associated memory. A priming injection of saline (1 mL/kg, i.p.) and morphine (3 mg/kg, i.p.) was administered 10 min before the reinstatement tests 1 and 2, respectively. n = 7–8 per group; **p < 0.01, compared to the control group. Data are expressed as mean ± SEM.

Next, we evaluated whether the intervention of berberine during the critical 6 h time window occurring immediately after the extinction training (aka cue exposure) might indicate an interaction between the treatment and consolidation of the extinction memory. In this experiment, the berberine treatment was administered 1 h or 6 h after each extinction session in the free access CPP extinction paradigm (**Figure 3A**). Eight out of 30 mice were excluded from the study due to strong initial preference or CPP training failure. As shown in **Figure 3B**, significant difference was observed between the CPP scores of pre-conditioning and post-conditioning tests (t _21_ = 10.16, *p* < 0.001), and remaining 22 mice in three independent groups (n = 7, 8, and 7) were subjected to the berberine treatment following the extinction training. Animals in the berberine groups were treated with berberine (25 mg/kg, i.g.) at 1 h or 6 h after each extinction session, respectively; while the control mice were administered corn oil (1 mL/kg, i.g.) 1 h after each extinction session. A two-way ANOVA (mixed design) revealed a significant difference in the CPP scores (**Figure 3C**) across berberine treatment (F_(2,__19)_ = 4.171, *p* = 0.032), and extinction sessions (F_(5,__95)_ = 24.11, *p* < 0.001), but not in berberine treatment × extinction sessions interactions (F_(10,__95)_ = 0.396, *p* = 0.946). In the reinstatement session, analysis of repeated measures also demonstrated a significant difference in the CPP scores across reinstatement test phase (F_(1,__19)_ = 23.81, *p* < 0.001), berberine treatment (F_(2,__19)_ = 5.538, *p* = 0.013), and berberine treatment × reinstatement test phase interactions (F_(2,__19)_ = 4.209, *p* = 0.031). Bonferroni *post hoc* analysis indicated a significant difference in 1 h berberine sub-group (*p* < 0.01) but not in the 6-h berberine sub-group (*p* > 0.05), as compared to the control group (**Figure 3D**). Therefore, these results suggest that the administration of berberine during the sensitive time window occurring immediately after the exposure to drug cues can facilitate the extinction of drug-associated behavior seemingly by enhancement of extinction memory consolidation.

### The Effect of Berberine on Spontaneous Recovery of Morphine-Induced Original Drug-Associated Memory

**Figure 4:**
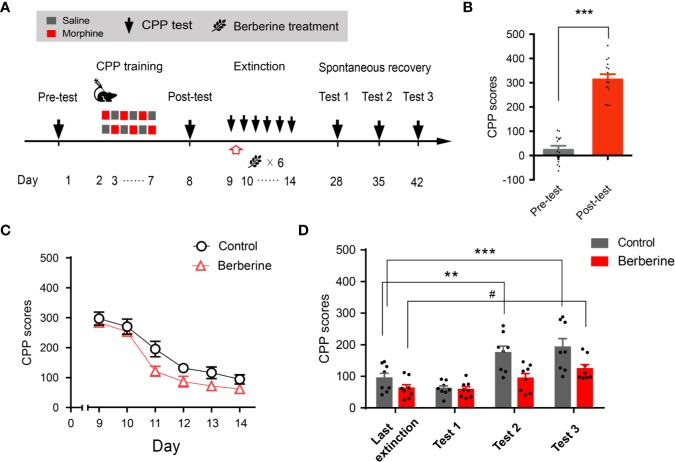
The effect of berberine on spontaneous recovery of morphine-induced original drug-associated memory **(A)** The experimental timeline of behavioral training and tests, and drug treatments **(B)** CPP scores of pre-test and post-test, which were conducted before and after CPP training. n = 16; ***p < 0.001, compared to the pre-test **(C)** Extinction of morphine-induced drug-associated behavior in a free access CPP extinction paradigm. Berberine (25 mg/kg, i.g.) was administered 1 h after each extinction training session **(D)** Spontaneous recovery of morphine-induced original drug-associated memory. All mice were kept in their home cages after completing the extinction sessions, and the spontaneous recovery tests were performed on days 28, 35, and 42. n = 7–8 per group; **p < 0.01, ***p < 0.001, compared to the CPP scores in the last extinction training session in the control group; ^#^*p* < 0.05, compared to the CPP scores in the last extinction training session in the berberine group. Data are expressed as mean ± SEM.

Extinction is believed to form a new conditioned stimulus-associative memory rather than to erase the original drug-associated memory (Liu et al., 2019). To elucidate whether the berberine treatment erased the morphine-induced drug-associated memory, we performed a spontaneous recovery test in a separate experiment (**Figure 4A**). Four out of 20 mice were excluded from the study due to strong initial preference or CPP training failure. Consistent with the above results, animals showed significant chamber preference after CPP conditioning (t _15_ = 13.98, *p* < 0.001; **Figure 4B**), and the berberine treatment facilitated the extinction of morphine-induced drug-associated memory (berberine treatment: F_(1,__14)_ = 12.35, *p* = 0.003; extinction sessions: F_(5,__70)_ = 54.12, *p* < 0.001; berberine treatment × extinction sessions interactions: F_(5,__70)_ = 0.8194, *p* = 0.540; **Figure 4C**). All mice were kept in their home cages after completing the extinction training, and spontaneous recovery tests were performed on Days 28, 35, and 42. Analysis by repeated measures revealed a significant difference in the CPP scores (**Figure 4D**) across berberine treatment (F_(1,__14)_ = 14.06, *p* = 0.002), and test phase (F_(3,__42)_ = 17.18, *p* < 0.001), but not in berberine treatment × test phase interactions (F_(3,__42)_ = 2.473, *p* = 0.075). Bonferroni *post hoc* analysis indicated a significant increase in CPP scores for the control group in Test 2 (*p* < 0.01, as compared to the CPP score on the last extinction session) and Test 3 (*p* < 0.001, as compared to the last extinction session). The CPP scores of berberine treated mice showed a significant increase only in Test 3 (*p* < 0.05, as compared to the last extinction session). Thus, the berberine treatment did not erase the original drug-associated memory, however, it delayed a spontaneous recovery of drug-associated memory.

### The Effect of Berberine on Extinction of Cocaine-Induced Drug-Associated Behavior

**Figure 5:**
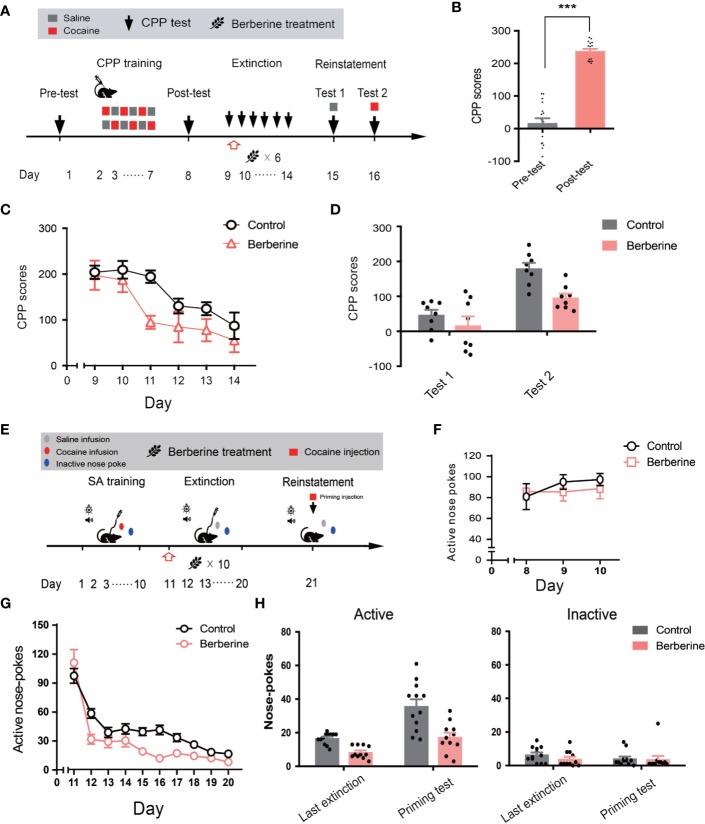
The effect of berberine on extinction of cocaine-induced drug-associated behavior in CPP and IVSA extinction paradigms **(A)** The experimental timeline of CPP training and tests, and drug treatments **(B)** CPP scores of pre-test and post-test. n = 16; ***p < 0.001, compared to the pre-test **(C)** Extinction of cocaine-induced drug-associated behavior in a free access CPP extinction paradigm. Corn oil (1 mL/kg, i.g.) and berberine (25 mg/kg, i.g.) were administered to mice in the control and berberine treatment groups, respectively, 1 h after each extinction training session **(D)** Reinstatement of cocaine-induced original drug-associated memory after CPP extinction. A priming injection of saline (1 mL/kg, i.p.) and cocaine (5 mg/kg, i.p.) was administered 10 min before the reinstatement tests 1 and 2, respectively **(E)** The experimental timeline of cocaine IVSA training and drug treatments **(F)** Active nose-pokes of last 3 days of IVSA training **(G)** Active nose-pokes during the extinction of cocaine IVSA. Corn oil (1 mL/kg, i.g.) and berberine (25 mg/kg, i.g.) were administered 1 h after each extinction training session **(H)** Active and inactive nose pokes in the reinstatement of cocaine IVSA. A priming injection of saline (1 mL/kg, i.p.) and cocaine (5 mg/kg, i.p.) was administered 10 min before the reinstatement test. n = 11–12 per group. Data are expressed as mean ± SEM. IVSA, intravenous self-administration.

Next, we investigated the effect of berberine on extinction of cocaine-induced drug-associated behavior using CPP (**Figure 5A**) and IVSA (**Figure 5E**) paradigms. During the extinction procedure, berberine (25 mg/kg, i.g.) or vehicle (corn oil, 1 mL/kg, i.g.) was administered 1 h after each CPP or IVSA extinction training session. Four out of 20 mice were excluded from the CPP experiment due to strong initial preference or training failure, and 16 remaining mice showed significant cocaine CPP in the post-conditioning test (t_15_ = 12.54, *p* < 0.001; **Figure 5B**). A two-way ANOVA (repeated measures) on CPP scores in extinction session (**Figure 5C**) revealed a significant difference across berberine treatment (F_(1,__14)_ = 6.484, *p* = 0.023), and extinction sessions (F_(5,__70)_ = 12.05, *p* < 0.001), but not in berberine treatment × extinction sessions interactions (F_(5,__70)_ = 1.074, *p* = 0.382). In the reinstatement session (**Figure 5D**), a separate ANOVA also showed a significant difference in the CPP scores across berberine treatment (F_(1,__14)_ = 8.290, *p* = 0.012), reinstatement condition (F_(1,__14)_ = 42.61, *p* < 0.001), but not in berberine treatment × reinstatement condition interactions (F_(1,__14)_ = 2.667, *p* = 0.125).

Furthermore, a total of 30 rats in 2 groups were trained to self-administer cocaine for 10 days. Seven animals were excluded from statistical analysis due to a loss of injection buttons or catheter failure. As shown in **Figure 5F**, active nose-pokes in the last 3 days of IVSA training indicated that all rats successfully acquired cocaine self-administration. For extinction responding (**Figure 5G**), a two-way ANOVA (repeated measures) revealed a significant difference in active nose-pokes across berberine treatment (F_(1,__21)_ = 8.816, *p* = 0.007), extinction sessions (F_(9,__189)_ = 72.59, *p* < 0.001), and berberine treatment × extinction sessions interactions (F_(9,__189)_ = 3.853, *p* < 0.001). For cocaine-primed reinstatement (**Figure 5H**), statistical analysis indicated a main effect of active nose-pokes across berberine treatment (F_(1,__21)_ = 24.72, *p* < 0.001), and reinstatement condition (F_(1,__21)_ = 30.17, *p* < 0.001), but not in berberine treatment × reinstatement condition interactions (F_(1,__21)_ = 3.889, *p* = 0.062). These results indicate that the berberine treatment significantly facilitated the extinction of cocaine-induced drug-associated behavior and prevented subsequent drug primed reinstatement of cocaine seeking.

### The Effect of Berberine Treatment on the BDNF, GluA1, and GluA2 Expression in the NAc After the Extinction Training Procedure

**Figure 6:**
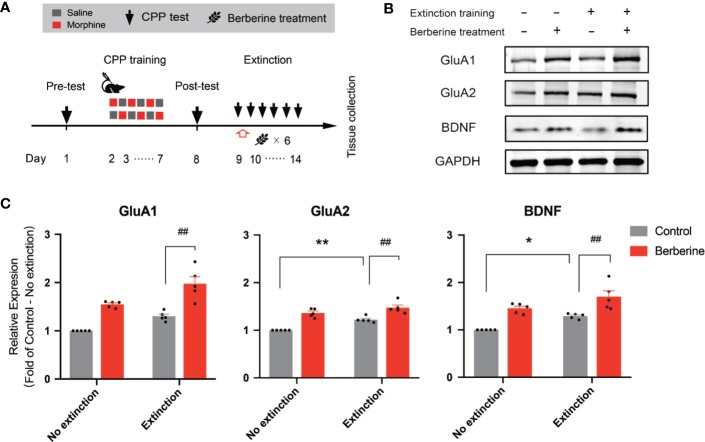
The effect of berberine treatment and extinction training on the expression of BDNF, GluA1, and GluA2 in the NAc **(A)** The experimental timeline of the CPP training and tests, drug treatments, and tissues collection **(B)** Representative image of GluA1, GluA2, and BDNF expression detected by Western blot analysis (for original full image see supplementary files, **Figure S2**) **(C)** The expressions of BDNF, GluA1, and GluA2 (fold of control—no extinction group) in the NAc. n = 5 per group. *p < 0.05, **p < 0.01, compared to the control—no extinction group; ^##^*p* < 0.01, compared to the control—extinction group. Data are expressed as mean ± SEM. BDNF, brain-derived neurotrophic factor; NAc, nucleus accumbens.

In this experiment, a total of 28 mice were assigned to four groups including no extinction training + vehicle treatment group, no extinction training + berberine treatment group, extinction training + vehicle treatment group, and extinction training + berberine treatment group to investigate the effect of berberine on the expressions of BDNF, GluA1, and GluA2 in the NAc after the CPP extinction. As shown in **Figure 6A**, CPP training and free access CPP extinction paradigm were performed as per the procedure described in *The Effect of Berberine on Extinction of Morphine-Induced Drug-Associated Behavior in a Free Access CPP Extinction Paradigm*, and the berberine (25 mg/kg, i.g.) or vehicle (corn oil, 1 mL/kg, i.g.) treatments were delivered 1 h after each extinction training session. Meanwhile, animals in the no extinction training groups received berberine or vehicle treatment once a day in their home cages. In the pre-conditioning and conditioning sessions, 7 out of 28 mice were excluded from the study due to strong initial preference or training failure. At the end of the experiment, five animals from each group were sacrificed 1 h after the last extinction training and drug treatment, and the NAc tissues were collected for GluA1, GluA2, and BDNF detection using Western blot analysis (**Figure 6B**). Separate two-way ANOVAs with berberine treatment and extinction training as between-subjects factor were used to analyze the data (**Figure 6C**), revealing significant main effects of berberine treatment and extinction training on GluA1 (berberine treatment: F_(1,16)_ = 61.89, *p* < 0.001; extinction training: F_(1,16)_ = 21.90, *p* < 0.001), GluA2 (berberine treatment: F_(1,16)_ = 71.57, *p* < 0.001; extinction training: F_(1,16)_ = 21.20, *p* < 0.001), and BDNF (berberine treatment: F_(1,16)_ = 39.76, *p* < 0.001; extinction training: F_(1,16)_ = 15.52, *p* = 0.001), however, no significant effect of berberine treatment × extinction training interactions (GluA1: F_(1,16)_ = 0.645, *p* = 0.434; GluA2: F_(1,16)_ = 2.347, *p* = 0.145; BDNF: F_(1,16)_ = 0.111, *p* = 0.743). These results indicate that the berberine treatment as a post-extinction intervention can potentiate extinction training-related changes in GluA1, GluA2, and BDNF expression in the NAc.

## Discussion

Our results indicate that extinction training combined with the berberine treatment delivered during the critical time window of memory consolidation facilitates the extinction of morphine- and cocaine-induced drug-associated behavior. We also demonstrated that the berberine treatment as a post-extinction intervention could significantly prevent reinstatement and spontaneous recovery of original drug-associated memories. Furthermore, we showed that the potential mechanisms underlying the therapeutic effects of berberine were associated with enhanced extinction memory consolidation and upregulation of plasticity-related proteins in the NAc, including BDNF, GluR1, and GluR2. Altogether, these results may pave a way for the development of effective berberine-based post-extinction pharmacological interventions for drug relapse prevention.

Extinction training forms new no drug/drug cue associative memories resulting in suppression of original memories (Millan et al., 2011). A growing number of studies have shown that pre- or post-extinction pharmacological interventions with gamma-aminobutyric acid (GABA) B and cholinergic receptor agonists can alter drug reward memories (Heinrichs et al., 2010; Stoll et al., 2018). However, most of the pharmacological agents tested in experimental studies are not suitable for human use due to their health-related side effects. Berberine is a popular OTC medication in China and other countries and has shown outstanding benefits in well-controlled clinical trials (Lahiri and Dutta, 1967; Gupte, 1975; Mohan et al., 1982). In addition, preclinical studies have reported that administration of berberine alleviates morphine withdrawal-associated depression- and anxiety-like behaviors as well as it reduces the acquisition and reinstatement of morphine-induced CPP (Lee et al., 2012; Vahdati Hassani et al., 2016). In the study by Hassani et al., a single berberine dose administered before a reinstatement test was sufficient to reduce reinstatement of morphine-induced CPP, and this effect was associated with inhibition of dopamine (DA) and N-methyl-d-aspartate (NMDA) systems. However, more studies are needed to elucidate the exact mechanisms underlying therapeutic benefits of berberine and explore its potential application in clinical settings.

In this study, we found that extinction training combined with the post-extinction berberine treatment facilitates the extinction of morphine-induced drug-associated behavior in free access and confined CPP extinction paradigms, as well as it reduces drug-primed reinstatement of drug-seeking. Our results also suggest the interaction of the post-extinction berberine intervention with consolidation of extinction memory during the critical 6 h time window (Xue et al., 2012; Luo et al., 2015) and suggest that the berberine treatment administered 6 h after the extinction training procedure (aka outside the critical time window) has no therapeutic benefits. Notably, substantial evidence from rodent and human studies has shown that extinguished drug-associated memories often return with the passage of time or after the exposure to drugs or drug cues. Therefore, we examined whether or not the berberine treatment could erase original drug-associated memories, using spontaneous recovery tests performed 14, 21, and 28 days after the last extinction training session. We found that the berberine treatment did not erase the original drug-associated memories; however, it delayed spontaneous recovery of morphine-induced original drug-associated memories. Though opioids and psychostimulants induce different behavioral changes through distinct neurobiological mechanisms (Badiani et al., 2011), our results indicate that the berberine treatment can facilitate the extinction of both morphine- and cocaine-induced CPP. Moreover, the berberine treatment significantly promoted extinction and inhibited reinstatement of cocaine-induced operant IVSA behavior, suggesting that berberine as a post-extinction intervention agent has therapeutic potential in relapse prevention. However, some issues should be considered with regard to interpretation of the present results. It is well known that extinction-related neuroadaptations within extinction circuits may erase the original memory trace (Centonze et al., 2005; Quirk et al., 2010). Although we attribute the function of berberine to the consolidation of extinction memory, a role of berberine in the process of original memory erasure requires further study. In addition, the effects of berberine on cocaine-induced CPP and IVSA behaviors seem to be less robust, particularly in animals with a lower extinction baseline. Therefore, it is possible that reinstatement responses of low magnitude were related to the low extinction baseline.

Numerous studies have elucidated the mechanisms underlying the extinction of drug-associated memories (McNally, 2014). One key target of interests is AMPA receptors that play an important role in persistence of memory across time, and extinction of drug-associated memory (Watterson and Olive, 2013; Trent et al., 2017; Chai et al., 2018). Several manipulations, including GluR2 overexpression and NMDA receptor partial agonist d-cycloserine administration, effectively promote extinction of drug seeking and prevent relapse (Sutton et al., 2003; Torregrossa et al., 2010). Furthermore, BDNF is a well-established molecular mediator of multiple stages of memory, and many drugs of abuse are known to alter the BDNF expression in neural circuits responsible for addictive behaviors (Barker et al., 2015). A recent study has shown that BDNF might be a downstream mechanism by which AMPA activation facilitates extinction of cocaine seeking (Quintero, 2013). Given the importance of BDNF and AMPA receptors in the extinction of drug memories, we examined the effect of berberine on the expression of BDNF and AMPA receptors in post-extinction training animals. Our results indicate that the extinction training increases the expressions of BDNF, GluR1, and GluR2 in the NAc and that post-extinction berberine alone was sufficient to increase the expression of these molecules and to further potentiate the extinction process. However, it is worthy to note that berberine increased the protein expressions regardless of the extinction training.

Although we have demonstrated that berberine facilitates the extinction of drug-associated behavior by enhancing consolidation of extinction memory, further studies are required to explore potential direct targets of berberine. A number of CNS diseases have been linked to abnormalities of gastrointestinal tract and recent studies suggest that gut microbiota plays a key role in the development of drug addiction (Kiraly et al., 2016; Ning et al., 2017; Meckel and Kiraly, 2019). Moreover, remarkable effects of berberine on gut microbiota and regulation of related disease pathophysiology have been recently reported (Cui et al., 2018; Feng et al., 2019; Yue et al., 2019). We, therefore, speculate that the effects of berberine on the extinction of drug-associated behavior might be related to its interaction with gut microbiota. In our next study, we plan to examine differences in gut microbiota in berberine- and non-berberine treated mice as we hypothesize that these differences might be associated with differences in relapse propensity. Furthermore, a number of studies found that berberine has multifunctional properties and can inhibit the activity of important pathogenic enzymes, ameliorate intracellular oxidative stress, attenuate neuroinflammation, trigger autophagy, and protect neurons against recognition memory damage (de Oliveira et al., 2019; Yuan et al., 2019; Zhao et al., 2019). Undeniably, more research is needed to understand the effects of pre-extinction berberine intervention on relapse and uncover the underlying neurochemical and molecular mechanisms. Given that berberine alone does not produce conditioned place preference or conditioned place aversion (Vahdati Hassani et al., 2016), the berberine treatment administered after the extinction training sessions, is unlikely to exert rewarding or adverse effects on its own. Therefore, it is unlikely that berberine facilitates the extinction of CPP or operant responding by any adverse effects. In support of this premise we found no effects of berberine treatment on drug-induced locomotor, as assessed during morphine-primed CPP reinstatement tests (see supplementary files, **Figure S1**).

In summary, the present study shows that berberine administered as a post-extinction pharmacological intervention significantly facilitates the extinction of morphine- and cocaine-induced drug-associated behavior and increases the levels of plasticity-related proteins: BDNF, GluR1, and GluR2 in the NAc. Given that the berberine treatment administered during the critical time window immediately after the extinction training (aka drug cue exposure) facilitates the extinction of CPP and drug seeking, berberine is an attractive pharmacotherapeutic candidate that might potentially mitigate risks of relapse in a clinical population.

## Data Availability Statement

The raw data supporting the conclusions of this article will be made available by the authors, without undue reservation.

## Ethics Statement

The animal study was reviewed and approved by Local Committee on Animal Care and Use and Protection of the Hebei Medical University.

## Author Contributions

DW, YL, CM, and BC conceived this work. DW and YL wrote, and EG revised the manuscript. XS, RH, and SF performed the animal experiments. HY performed the western blot, BX and HB analyzed and interpreted the data. All authors read, revised, and approved the manuscript.

## Funding

This work was supported by the College Students Innovative Pilot Project of Hebei Medical University (No. USIP2018014, No. USIP2017018), and the National Natural Science Foundation of China (No. 81772019).

## Conflict of Interest

The authors declare that the research was conducted in the absence of any commercial or financial relationships that could be construed as a potential conflict of interest.

## Supplementary Material

Figure S1 Locomotion of the mice (distance traveled) in morphine-primed reinstatement tests after free access **(A)** and confined **(B)** CPP extinction training procedure. One-way ANOVA revealed no significant difference between the groups (A: F_(3,28)_ = 0.8207, *p* = 0.4935; B: F_(3,27)_ = 0.5396, *p* = 0.6592).

Figure S2 Original full image of Western blot for detecting the levels of GluA1 **(A)**, GluA2 **(B)**, and BDNF **(C)**. The first lane is a marker, the second lane denotes lysate of NAc tissue from naive mice as a control. The molecular weights of GluA1, GluA2, BDNF, and GAPDH approximately are 100, 100, 15, and 36 kDa, respectively.

## Abbreviations

AMPA, α-amino-3-hydroxy-5-methyl-4-isoxazole propionic acid; Amy, amygdala; ANOVA, analysis of variance; BDNF, brain-derived neurotrophic factor; CNS, central nervous system; CPP, conditioned place preference; DA, dopamine; FR 1, Fixed-ratio 1; GAPDH, Glyceraldehyde 3-phosphate dehydrogenase; IVSA, intravenous self-administration; NAc, nucleus accumbens; NMDA, N-methyl-d-aspartate; OTC, over the counter; PFC, prefrontal cortex; TBS, tris-buffered saline.
