# Supplementary figures and images for: Berberine Facilitates Extinction of Drug-Associated Behavior and Inhibits Reinstatement of Drug Seeking
Source: Front Pharmacol. 2020 Apr 24;11:476. doi: 10.3389/fphar.2020.00476 (PMC7194034; doi:10.3389/fphar.2020.00476)

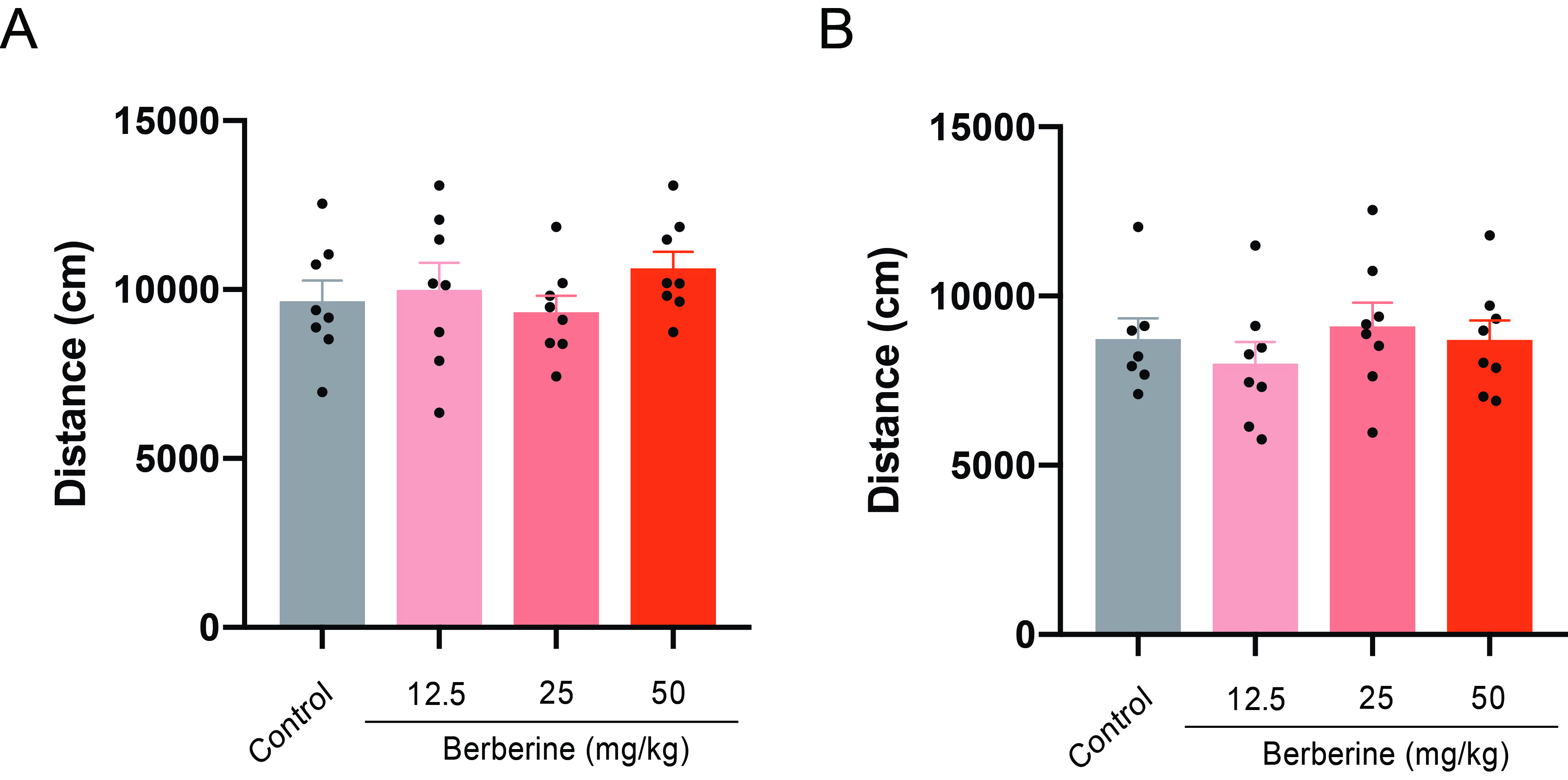

Supplement: Figure S1 — Locomotion of the mice (distance traveled) in morphine-primed reinstatement tests after free access (A) and confined (B) CPP extinction training procedure. One-way ANOVA revealed no significant difference between the groups (A: F(3,28) = 0.8207, p = 0.4935; B: F(3,27) = 0.5396, p = 0.6592). [file Image_1.jpeg]

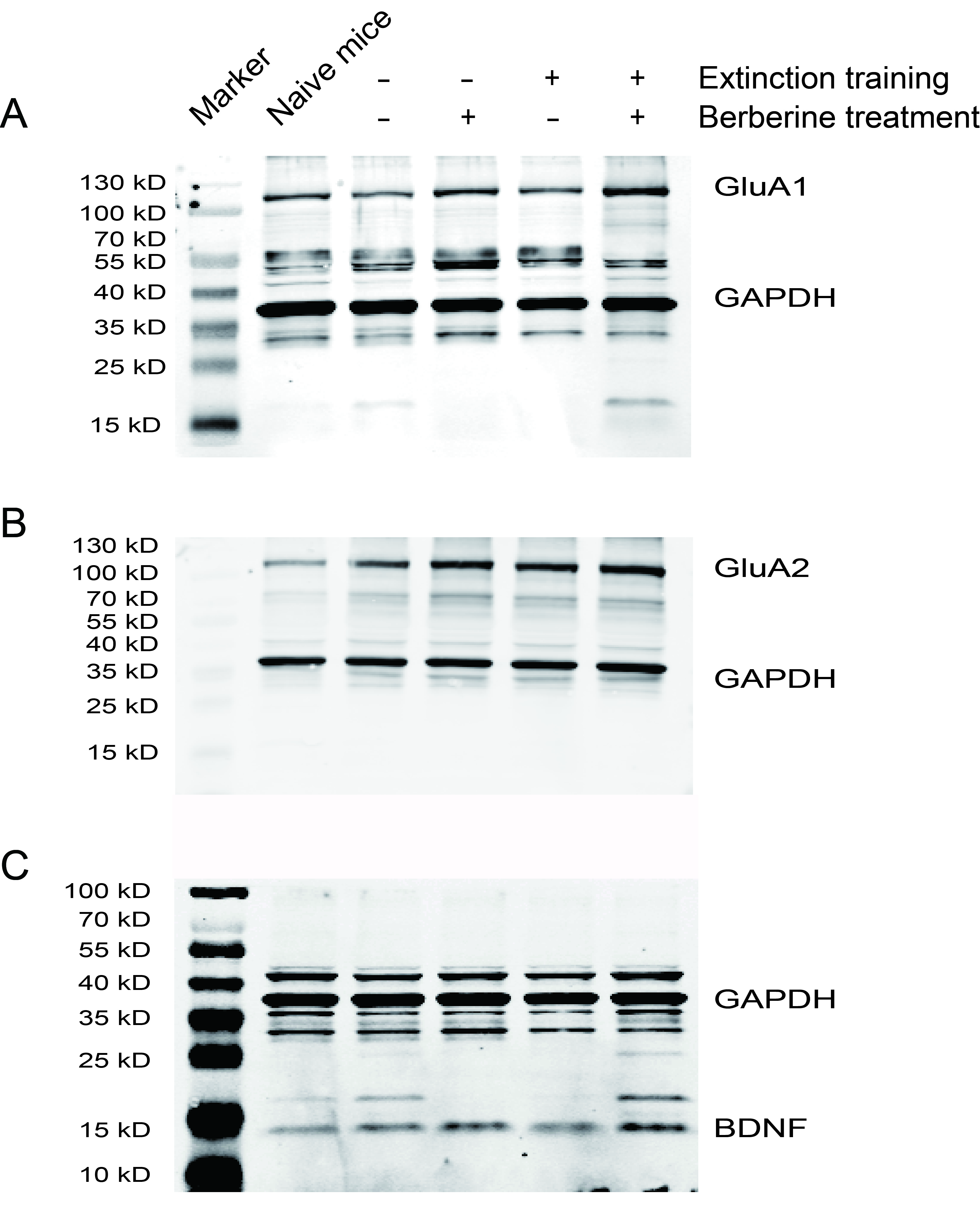

Supplement: Figure S2 — Original full image of Western blot for detecting the levels of GluA1 (A), GluA2 (B), and BDNF (C). The first lane is a marker, the second lane denotes lysate of NAc tissue from naive mice as a control. The molecular weights of GluA1, GluA2, BDNF, and GAPDH approximately are 100, 100, 15, and 36 kDa, respectively. [file Image_2.jpeg]
